# Supplementary material for: Improved prediction of MAPKi response duration in melanoma patients using genomic data and machine learning
Source: NPJ Precis Oncol. 2025 Jul 9;9:231. doi: 10.1038/s41698-025-00814-y (PMC12241329; doi:10.1038/s41698-025-00814-y)
Supplement: Supplementary file 1 — Supplementary Information [file 41698_2025_814_MOESM1_ESM.pdf]

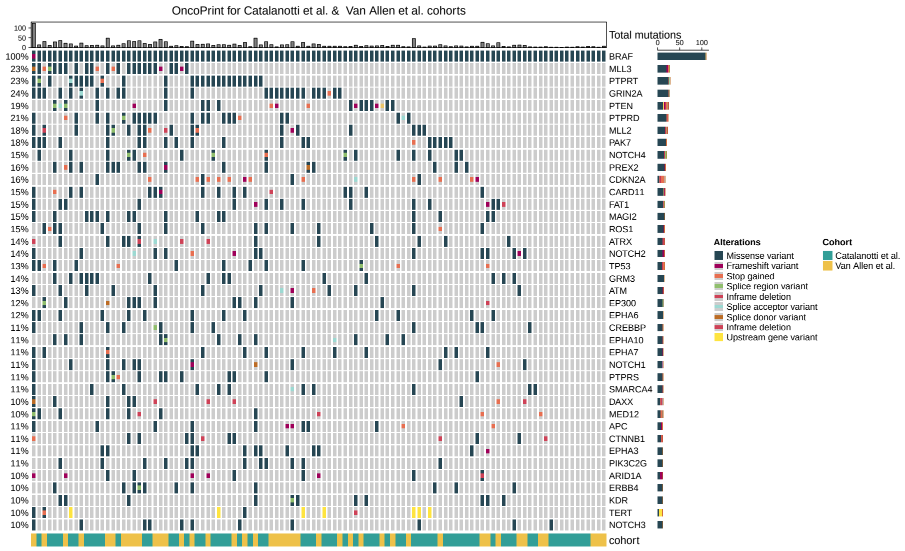

Supplementary Figure 1: **Oncoplot from Catalanotti *et al.* and Van Allen *et al.* cohorts**. Genetic alterations over the common number of sequenced genes among the two cohorts (275 genes). The Oncoplot has been produced using the ComplexHeatmap package on R studio. Rows represent specific genes and columns represent individual patients. Only genes with at least 10% of mutation prevalence are indicated. The total number of mutations per patient is shown in the top annotation bar plot.

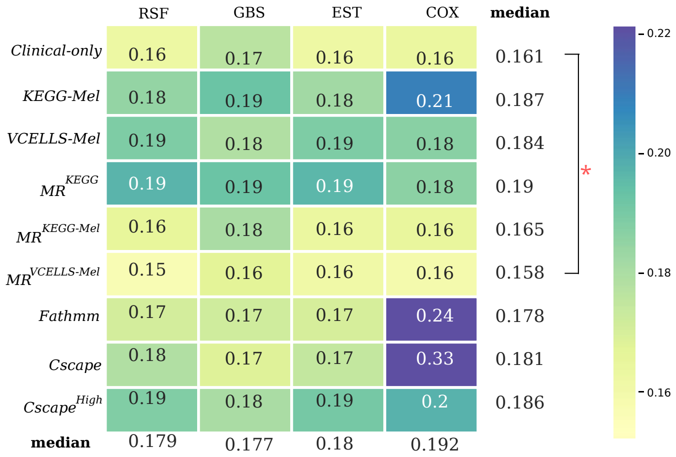

Supplementary Figure 2: **Integrated Brier scores.** Heatmap showing the integrated brier score of each dataset/algorithm pair. The median integrated Brier score was calculated from the Brier scores obtained during the testing procedure. Values are represented as a color gradient according to the distribution of the obtained values (0.161- 0.34). The median performance values for each dataset with all algorithms and for each algorithm using all datasets are shown in the last column and last row, respectively. The Wilcoxon signed-rank test was used for datasets comparison ; *p-value<0.05 versus the *Clinical-only* dataset.

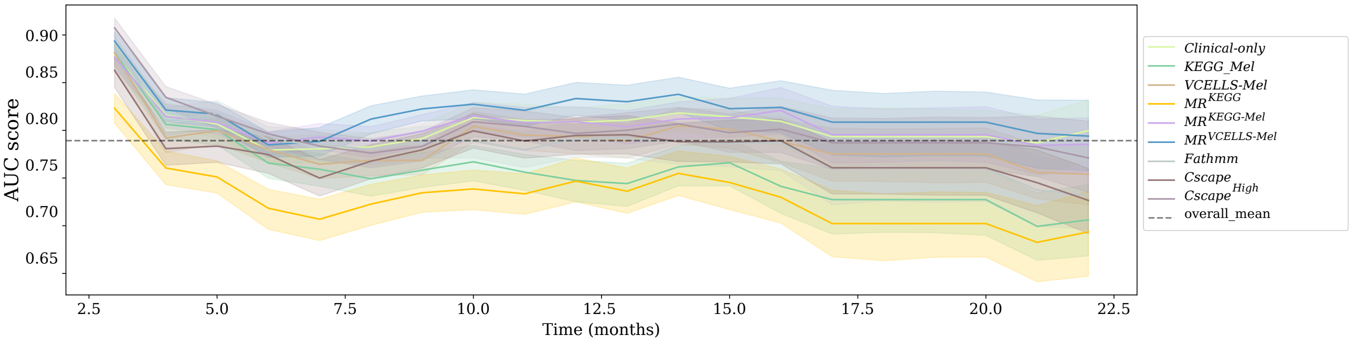

Supplementary Figure 3: **Evaluation of model predictions over time (Time-dependent AUC)** Comparison of the performance of the feature datasets for all algorithms over 22 months. Each line refers to the mean AUC value of that feature datasets at each time point with their associated standard deviation.

Dataset	Description	Feature composition	Total # feature
Clinical-only	Clinical features	8	8
KEGG-Mel	Clinical features + KEGG melanoma network data	8+70	78
VCELLS-Mel	Clinical features + VCELLs melanoma network data	8+119	127
MR^{KEGG}	Clinical features + All pathway probabilities (KEGG+VCELLs)	8+332	340
$MR^{KEGG\text{-}Mel}$	Clinical features + KEGG melanoma pathway probabilities	8+1	9
$MR^{VCELLS\text{-}Mel}$	Clinical features + VCells melanoma pathway probabilities	8+1	9
FATHMM	Clinical features + fathmm data (5% threshold)	8+39	47
Cscape	Clinical features + Cscape data (5% threshold)	8+53	61
$Cscape^{High}$	Clinical features + Cscape data (only high confidence genes)	8+171	179

Supplementary Table 1: Description of the dataset composition.

Genes	
APC	PTPRS
ATM	PTPRT
BRAF	ROR2
CBL	ROS1
CBLB	TP53
CDKN2A	TP63
CTNNB1	
EGFR	
EPHA10	
EPHA3	
EPHA5	
EPHA7	
EPHB4	
EPHB6	
ERBB3	
ERBB4	
FGFR2	
FGFR4	
FLT3	
FLT4	
GNAS	
GRIN2A	
KDR	
NF2	
NOTCH2	
NTRK1	
NTRK3	
PIK3C2G	
PIK3CA	
PIK3CG	
PTCH1	
PTEN	
PTPRD	

Supplementary Table 2: List of genes for *Fathmm* dataset.

Genes	
ALK	MLL2
APC	MLL3
ARHGAP26	NOTCH1
ARID1A	NOTCH2
ARID2	NOTCH4
ATM	NTRK3
BAP1	PAK7
BRAF	PIK3C2G
CARD11	PIK3CA
CDKN2A	PRDM1
CREBBP	PREX2
CTNNB1	PTEN
DAXX	PTPRD
DICER1	PTPRS
EPHA10	PTPRT
EPHA3	RB1
EPHA4	ROS1
EPHA5	SMARCA4
EPHA6	TP53
EPHA7	TP63
EPHB1	
EPHB2	
EPHB3	
ERBB4	
FAT1	
FLT4	
GRIN2A	
GRM3	
KDR	
MAGI2	
MAP2K1	
MET	
MLL	

Supplementary Table 3: List of genes for *Cscape* dataset.

Genes					
ABL1	CYLD	FH	MDM2	PIK3R2	TMPRSS2
ABL2	DAXX	FLCN	MDM4	PKM2	TNFAIP3
AKT1	DDR2	FLT1	MED12	PPP2R1A	TP53
AKT2	DICER1	FLT3	MEN1	PRDM1	TP63
AKT3	DIS3	FLT4	MET	PREX2	TSC1
ALK	DNMT1	GLI1	MLL	PRKAA2	TSC2
APC	DNMT3A	GLI3	MLL2	PRKCI	
ARHGAP26	EGFR	GRIN2A	MLL3	PTCH1	
ARID1A	EP300	GRM3	MPL	PTEN	
ARID2	EPHA10	GSK3B	MTOR	PTPN11	
ATM	EPHA2	HDAC2	MYC	PTPRD	
ATRX	EPHA3	HIF1A	MYCN	PTPRS	
BAP1	EPHA4	HNF1A	NCOA2	PTPRT	
BCL2L1	EPHA5	IDH1	NF1	RAF1	
BCL2L11	EPHA6	IGF1R	NFKB1	RARA	
BCL6	EPHA7	IKZF1	NFKB2	RB1	
BRAF	EPHB1	IL7R	NOTCH1	RICTOR	
BRCA1	EPHB2	INPP4A	NOTCH2	RNF43	
BUB1B	EPHB3	INPP4B	NOTCH3	ROR2	
CARD11	EPHB4	INSR	NOTCH4	ROS1	
CBL	EPHB6	JAK1	NRAS	RPTOR	
CBLB	ERBB4	JAK2	NTRK2	SETD2	
CDC73	ERG	JUN	NTRK3	SF3B1	
CDH1	ESR1	KCNJ5	PAK7	SMAD4	
CDH11	ETV1	KDR	PARK2	SMARCA4	
CDK12	ETV6	KLF6	PARP1	SMARCB1	
CDK4	EZH2	LDHA	PBRM1	SPOP	
CDKN2A	FAS	LGR6	PDGFRA	SUFU	
CHEK1	FAT1	MAGI2	PDGFRB	TEK	
CIC	FBXO11	MAP2K1	PIK3C2G	TERT	
CREBBP	FBXW7	MAP2K2	PIK3CA	TET1	
CSF1R	FGFR2	MAP2K4	PIK3CG	TET2	
CTNNB1	FGFR4	MCL1	PIK3R1	TGFBR2	

Supplementary Table 4: List of genes for $Cscape^{High}$ dataset.

Genes	
AKT1	FGF9
AKT2	FGFR1
AKT3	GADD45A
ARAF	GADD45B
BAD	GADD45G
BAK1	HGF
BAX	HRAS
BRAF	IGF1
CCND1	IGF1R
CDK4	KRAS
CDK6	MAP2K1
CDKN1A	MAP2K2
CDKN2A	MAPK1
DDB2	MAPK3
E2F1	MDM2
E2F2	MET
E2F3	NRAS
EGF	PDGFA
EGFR	PDGFB
FGF1	PDGFC
FGF10	PDGFD
FGF16	PDGFRA
FGF17	PDGFRB
FGF18	PIK3CA
FGF19	PIK3CB
FGF2	PIK3CD
FGF20	PIK3R1
FGF21	PIK3R2
FGF22	PIK3R3
FGF23	POLK
FGF3	PTEN
FGF4	RAF1
FGF5	RB1
FGF6	TP53
FGF7	RB1
FGF8	TP53

Supplementary Table 5: List of genes for KEGG-melanoma pathway datasets

Genes			
ACTG1	FOS	MMP2	TIAM1
ADAM10	FOXO3	MMP3	TNF
ADAM17	GNA12	MYC	TP53
ADCY5	GNAS	NFATC3	UBE4B
AKT1	GNAT2	NOTCH2	USP29
ATM	GSK3A	NRAS	USP42
ATR	GSK3B	NUMB	USP7
BAD	GZMB	PAK3	VIM
BAK1	HGF	PDPK1	YWHAH
BAX	HGFAC	PLCG1	YY1
BCL2L11	HIF1A	PMAIP1	ZDHHC9
BCL3	HIPK2	PPP1CC	
BID	HNRNPK	PPP3CA	
BRAF	HPN	PRKAA1	
CASP3	HRAS	PRKCE	
CASP7	HTRA2	PRKCQ	
CASP8	ICMT	PSMD10	
CASP9	IQGAP1	PTEN	
CDC42	JUN	PTPN3	
CDH1	LIMK1	RAC1	
CFL2	MAP2K2	RAF1	
CHEK1	MAP2K4	RAP1B	
CHEK2	MAP2K7	RAPGEF1	
CREB1	MAP3K1	RB1	
CSNK2A1	MAP3K3	RCE1	
CTNNA1	MAP3K6	RPS6KA3	
CTNNB1	MAPK14	SKI	
CTNND1	MAPK3	SMAD2	
CYCS	MAPK8IP1	SOCS2	
CYLD	MAPKAPK2	SOS1	
CYR61	MARCKS	SPINT1	
DIABLO	MDM2	SPINT2	
DUSP7	MDM4	ST14	
EGLN2	MITF	STAT3	
EP300	MMP1	STK11	
EPAS1	MMP14	STRAP	

Supplementary Table 6: List of genes for VCELLS-melanoma pathway datasets

Features
sex [Male/Female]
age [continuous]
LDH [Normal/Elevated]
disease control rate [Complete response/Partial response/Stable disease/Progressive disease]
cobimetinib [Yes/No]
dabrafenib [Yes/No]
trametinib [Yes/No]
vemurafenib [Yes/No]

Supplementary Table 7: List of clinical features included in the datasets

Information	Values	Total	Van Allen et al.	Blateau et al.	Catalanotti et al.	Louveau et al.
Total number of patients	-	184	45	53	66	20
Treatment prior to MAPKi	no	158 (86 %)	45 (100 %)	50 (95%)	47 (72%)	16 (80%)
	yes	7 (4 %)	-	3 (5%)	-	4 (20%)
	yes - Chemotherapy	11 (6%)	-	-	11 (16%)	-
	yes - IL-2 (High dose IL-2)	4 (2.17 %)	-	-	4 (6%)	-
	yes - MEKi (MK-3475)	1 (0.5%)	-	-	1 (0.16%)	-
	yes - IFN	1 (0.5%)	-	-	1 (0.16%)	-
	yes - ipilimumab	2 (1%)	-	-	2 (3%)	-

Supplementary Table 8: Treatment regimens.
